# Eye-controlled endoscopy — a benchtop trial of a novel robotic steering platform — iGAZE2

**DOI:** 10.1007/s11701-024-02022-5

**Published:** 2024-06-25

**Authors:** Arun Sivananthan, Adrian Rubio-Solis, Ara Darzi, George Mylonas, Nisha Patel

**Affiliations:** 1 Imperial College NHS Healthcare Trust, London, W2 1NY UK; 2 The Hamlyn Centre for Robotic Surgery, Imperial College London, London, UK

**Keywords:** Eye tracking, Robotic endoscopy, Touchless interactions

## Abstract

The endoscopic control system has remained similar in design for many decades The remit of advanced therapeutic endoscopy continues to expand requiring precision control and high cognitive workloads. Robotic systems are emerging, but all still require bimanual control and expensive and large new systems. Eye tracking is an exciting area that can be used as an endoscope control system. This is a study to establish the feasibility of an eye-controlled endoscope and compare its performance and cognitive demand to use of a conventional endoscope. An eye gaze-control system consisting of eye-tracking glasses, customised software and a small motor unit was built and attached to a conventional endoscope. Twelve non-endoscopists used both the eye gaze system and a conventional endoscope to complete a benchtop task in a simulated oesophagus and stomach. Completion of tasks was timed. Subjective feedback was collected from each participant on task load using the NASA Task Load Index. Participants were significantly quicker completing the task using iGAZE2 vs a conventional endoscope (65.02 ± 16.34s vs 104.21 ± 51.31s, *p* = 0.013) Participants were also significantly quicker completing retroflexion using iGAZE2 vs a conventional endoscope (8.48 ± 3.08 vs 11.38 ± 5.36s, *p* = 0.036). Participants reported a significantly lower workload (raw NASA-TLX score) when using iGAZE2 vs the conventional endoscope (152.1 ± 63.4 vs 319.6 ± 81.6, *p* = 0.0001) (Fig. 7). Users found iGAZE2 to have a significantly lower temporal demand, mental demand, effort, mental demand, physical demand, and frustration level. The eye gaze system is an exciting, small, and retrofittable system to any endoscope. The system shows exciting potential as a novel endoscopic control system with a significantly lower workload and better performance in novices suggesting a more intuitive control system.

**Supplementary Information:**

The online version contains supplementary material available at 10.1007/s11701-024-02022-5.

## Introduction

Eye-tracking technology has great potential as a novel control system for luminal endoscopy. The control system of the endoscope has remained unchanged for several decades. The conventional endoscope is controlled by two wheels attached to antagonistic cables that steer the endoscope tip [1]. This steering mechanism in combination with the flexibility and looping of an endoscope makes conventional endoscopy a challenging skill to acquire with well documented poor ergonomics and a steep learning curve [2, 3].

The remit of luminal endoscopy continues to expand, nonetheless. Endoscopic submucosal dissection is a valuable technique allowing resection of complex lesions which may help avoid surgical colonic resection as well as reduce risk of recurrence vs endoscopic musical resection. Other third space endoscopic procedures such as Per Oral Endomyotomy (POEM) for achalasia are increasingly employed as a non-surgical management option [4]. These techniques require fine movement with small errors of margin between a successful procedure and a perforation [5, 6] and high demands on mental workload. Due to the fine bimanual control required, the endoscopist is also reliant on assistants to operate the various instruments required during the therapeutic endoscopy.

There have been several novel robotic endoscopic platforms proposed and trialled to facilitate intuitive endoscope control. All these systems are reliant on manual control platforms which are predominantly joystick based. Those focussed on advanced therapy have novel control systems to both allow intuitive steering of the endoscope and control of endoscopic instruments such as dissection knives. These systems are expensive with large footprints, are bimanually controlled and in some cases require more than one operator [7].

Eye tracking of endoscopist’s gaze patterns is an increasingly studied area providing valuable insights into the visual gaze patterns most suited to lesion detection as well as being used as an objective tool to compare new endoscopy-imaging technologies [8]. We have previously presented a novel gaze-controlled endoscope utilising gaze tracking as a control system rather than a research tool. Using eye-tracking glasses and a motorised system the endoscope tip follows the direction of the endoscopist’s gaze. In the previous system head tilting, and forward and backward movement by the user was able to rotate and insert and withdraw the scope. The initial gaze system was entirely hands free [9].

The system has undergone multiple reiterations to produce the updated and more ergonomic system: iGAZE2. The system now has gaze-controlled tip steering, but insertion and withdrawal are performed with the right hand. This system has a significantly smaller footprint with a miniaturised motor system with low-cost components that can be easily fitted to any conventional endoscope.

In this study, we trial the new system (iGAZE2) with novices to assess the feasibility, intuitiveness, and workload of this system vs a conventional endoscope.

## Methodology

### Robotic control system

**Fig. 1:**
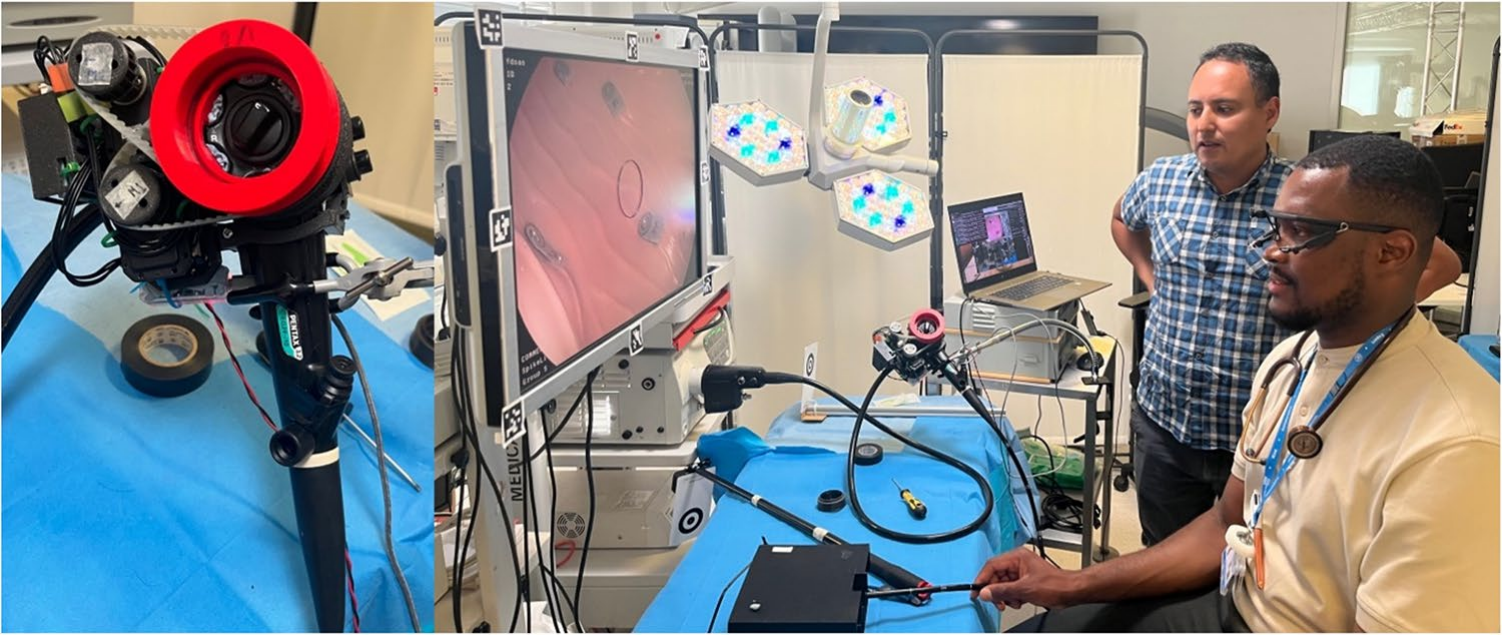
The iGAZE2 system, with small mount for endoscope (left) and full setup (right)

iGAZE2 (Fig. 1) is a new iteration of a previous robotic system described elsewhere [9] designed to have a smaller footprint, be cheaper and remain retrofittable to any existing endoscope.

This system consists of 3D-printed gears placed over the endoscope steering wheels. These gears were controlled by two small motors (Dynamixel RX-24F, Robotis, Korea) with a USB input to receive input signals. This system was mounted in a small unit attached to the handle of a Pentax EG29-i10 Video Gastroscope. This lightweight small mount allows access to all buttons and valves of the endoscope and rapid detachment of the system if required.

### Gaze control

Endoscope tip control is based on the direction of the users gaze in relation to the screen. The user wore eye-tracking glasses (Pupil Core, Pupil Labs, Berlin, Germany). These eye-tracking glasses, weighing only 22.75g have two cameras recording the pupils and one camera recording the user’s point of view. Following a simple calibration these eye-tracking glasses using two small cameras facing the pupils and one facing outwards are accurately able to measure the point of binocular gaze relative to the centre of the 42-inch 1080p LG endoscope monitor (LG electronics, Seoul, South Korea). Using a custom gaze contingent framework developed by Kogkas et al. [10, 11] the point of gaze relative is translated into an x and y co-ordinate relative to the centre of the screen. This is translated into a predicted x and y directed motor activation of the endoscope steering wheels. The closed loop allows continuous correction relative to the point of gaze and the centre of screen allowing rapid accurate steering. The motos is set to 15 N-m at 15 revolutions per minute.

### User interface

**Fig. 2:**
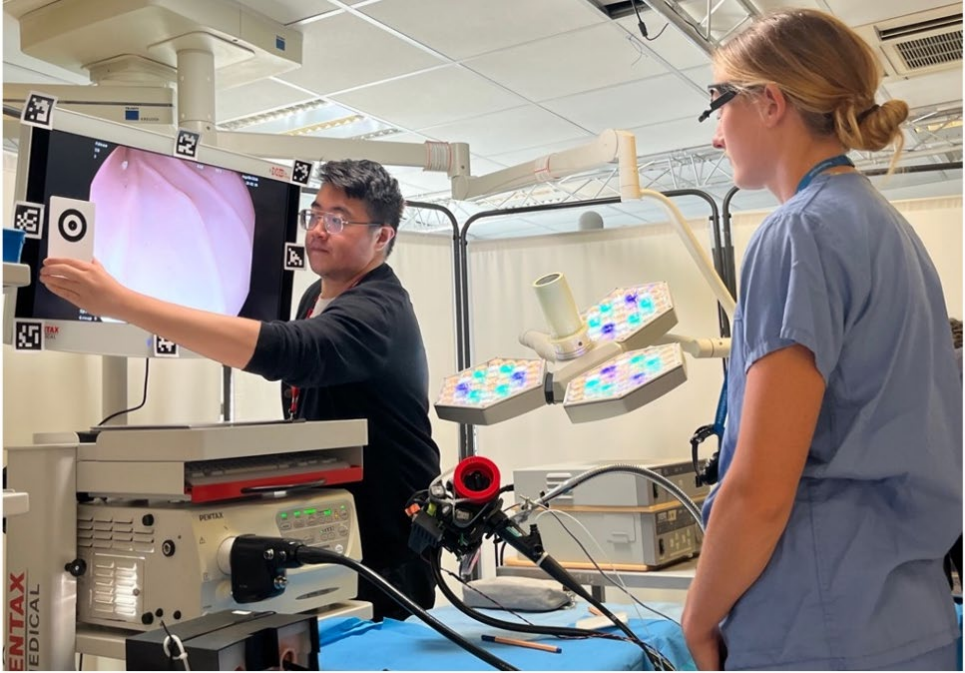
Eye-tracking glasses (left) (Pupil Core, Pupil Labs, Berlin, Germnay) and calibration (right)

The user sat or stood 2 m away from the monitor with the monitor placed slightly below the endoscopists eye line as reported to be the most ergonomically favourable position. A quick calibration was performed requiring the user to trace nine targets around the outside perimeter and centre of the monitor (Fig. 2). The user was able to insert and withdraw the scope manually with one hand. Steering was controlled by the direction of gaze. For example, if the user looked to the top right of the monitor the endoscope tip would move so the point of focus was now central on the monitor screen. A foot pedal was added to allow automatic retroflexion following deployment of the pedal for two seconds.

## Study design

This study was a controlled trial to compare iGAZE2 to a conventional endoscope in novices, in terms of performance and user workload.

Twelve participants with no previous endoscopy, or gaze-control experience were recruited. The age range of the group was 24 – 38 years. The group consisted of doctors (foundation trainees, an internal medical trainee and a microbiologist), engineers, non-clinical scientists and an advertising executive.

Participants were randomised to begin on either the gaze-control system or the conventional endoscope. Each participant was given a standardised 2 min instruction by the author AS on the system they were first using and a standardised explanation of the benchtop task.

**Fig. 3:**
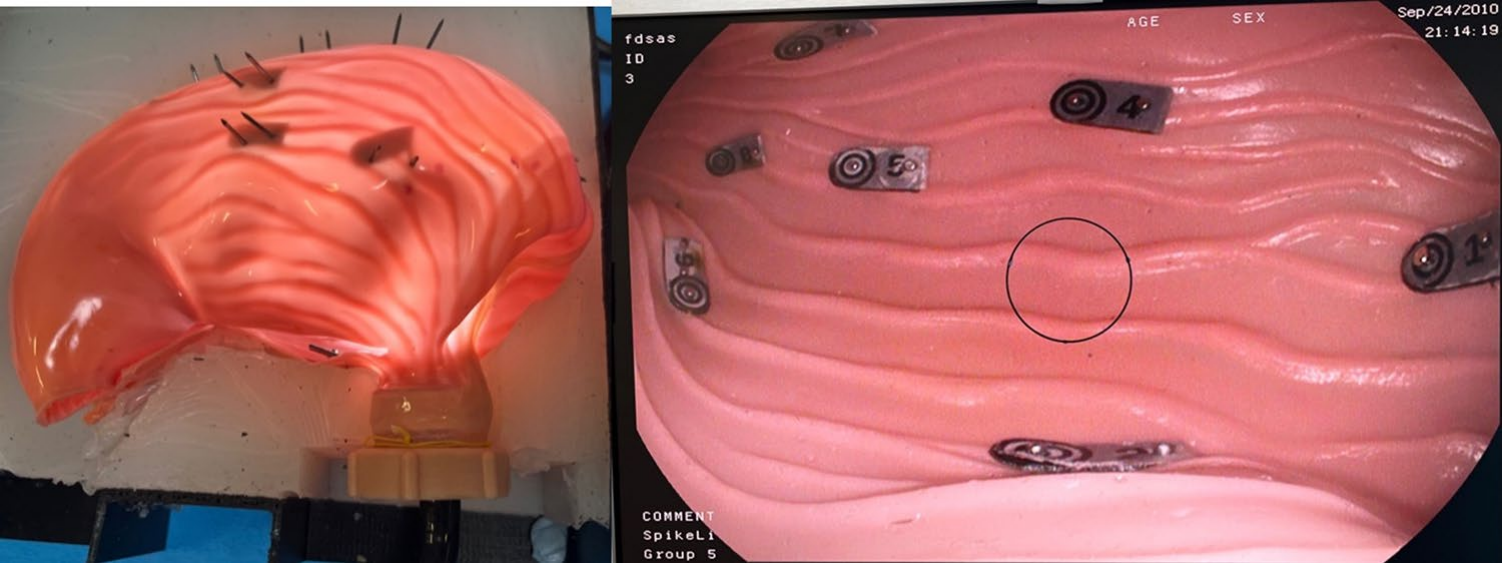
Anatomical stomach with box removed (left), monitor view of stomach model with targets (right)

The benchtop task consisted of an anatomically accurate model of the stomach in a closed black box (Fig. 3). Within the stomach nine numbered targets were placed at various points including fundus, body and pylorus. The participant was then required to complete retroflexion to visualise the gastric cardia.

Each task began with the endoscope tip placed at the ‘gastro-oesophageal junction’. The endoscopist was then required to locate the numbered targets from 1 to 9 in sequential order by placing the target in the central view. The central view was delineated with a 4cm diameter circle placed in the centre of the monitor. Participants were required to place the targets in the central view and ‘en face’.

Each participant completed the task five times and then transferred to the alternate system with a standardised instruction on the second system prior to completing the task five times again. Times were recorded and subjective feedback for each system was acquired directly after completing the task with each system to avoid recall bias.

## Outcomes

### Objective outcomes

The total time to find all targets, the time to complete retroflexion and the total time of each task was recorded.

### Subjective feedback

Subjective feedback on the workload of each system was collected using the raw NASA-TLX score {Citation}. The NASA-TLX score has been validated in assessing workload in both diagnostic and therapeutic endoscopy [12, 13]. This is a ten-point analogue scale validated in both diagnostic and therapeutic endoscopy. The NASA-TLX score domains included mental demand, physical demand, temporal demand, effort, and frustration. The domain ‘performance’ was removed as this was measured objectively. The data was collected with the official NASA-TLX ios (iphone operating system) application.

### Data analysis

All times were recorded as mean ± standard deviation to 2 decimal places. Total task times were taken as an average of the five times recorded per system. NASA-TLX scores were reported to 1 decimal place.

All comparisons were conducted using within subject analysis. The following comparisons were undertaken:

- task-completion time of iGAZE2 vs conventional control;
- retroflexion time of iGAZE2 vs conventional control;
- total NASA-TLX scores of iGAZE2 vs conventional control;
- NASA-TLX subdomain scores (mental demand, physical demand, temporal demand, effort and frustration) of iGAZE2 vs conventional control.

The paired t test with two tails was used to compare the normally distributed groups. A *p* value below 0.05 was used to denote significance.

## Results

### Completion times

**Fig. 4:**
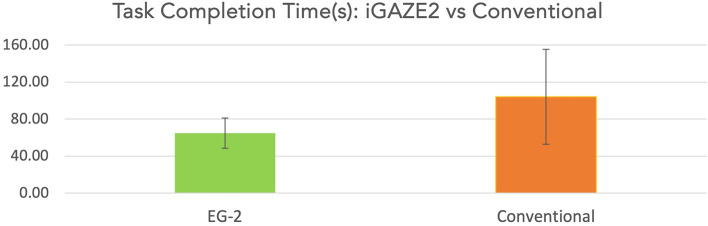
Task-completion time in seconds of iGAZE2 vs conventional (with standard deviations)

Participants were significantly quicker completing the task using iGAZE2 vs a conventional endoscope (65.02 ± 16.34s vs 104.21 ± 51.31s, *p* = 0.013) (Fig. 4).

**Fig. 5:**
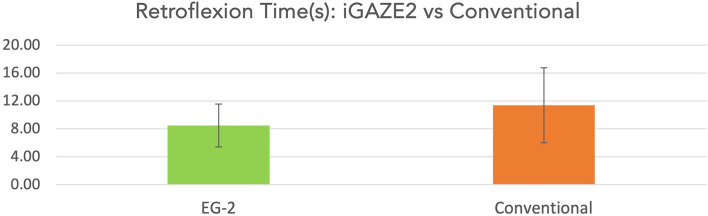
Retroflexion time in seconds of iGAZE2 vs conventional (with standard deviations)

Participants were also significantly quicker completing retroflexion using iGAZE2 vs a conventional endoscope (8.48 ± 3.08 vs 11.38 ± 5.36s, *p* = 0.036) (Fig. 5).

### Workload scores

**Fig. 6:**
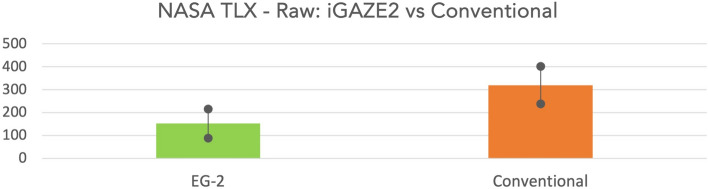
NASA-TLX score of iGAZE2 vs conventional (with standard deviations)

Participants reported a significantly lower workload (raw NASA-TLX score) when using iGAZE2 vs the conventional endoscope (152.1 ± 63.4 vs 319.6 ± 81.6, *p* = 0.0001) (Fig. 6).

**Fig. 7:**
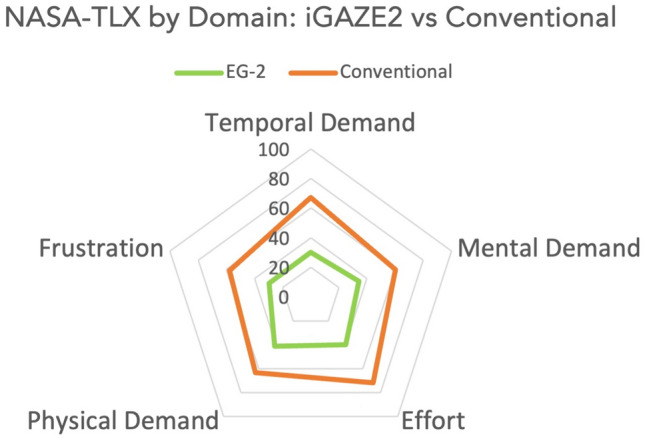
Radar plot of NASA-TLX subdomains of iGAZE2 vs conventional (with standard deviations)

For all subdomains users found iGAZE2 to have a lower temporal demand: (30.0 ± 14.0 vs 67.1 ± 15.9, *p* = 0.0001), mental demand (34.2 ± 16.6 vs 60.0 ± 26.1, *p* = 0.0165), effort(40.0 ± 19.8 vs 71.7 ± 16.3, *p* = 0.0006), mental demand (34.2 ± 16.6 vs 60.0 ± 26.1, *p* = 0.0165), physical demand (24.6 ± 20.9 vs 63.3 ± 26.0, *p* = 0.0001)and frustration (27.9 ± 20.3 vs 57.5 ± 21.1, *p* = 0.0079) (Fig. 7).

## Discussion

We present the trial data of iGAZE2, an updated eye-controlled robotised endoscope control system. The previous trial using the original system proved the feasibility of an eye-controlled robotised endoscope control system. The trial also suggested the system may be more intuitive but was not designed to answer this question. The trial design of this new study used only novices and allowing us to ascertain the comparative intuitiveness of iGAZE2 vs conventional endoscopy through both objective and subjective measures.

All users were significantly quicker using iGAZE2 suggesting that iGAZE2, a gaze-controlled system was more intuitive denoted by the improved performance. The addition of a foot pedal also proved to be a more intuitive method to control retroflexion with significantly quicker completion of retroflexion with iGAZE2.

All users reported a significantly lower workload across all domains suggesting further the intuitiveness of iGAZE2 vs conventional endoscopy. In the previous trial this has been true other than for physical demand. The previous system used head tilting and body movement to control rotation and insertion of the endoscope. This was removed in iGAZE2 with insertion and withdrawal controlled by the right hand. This update seemed to improve the workload further with a significant lower physical demand in iGAZE2 vs conventional endoscopy.

The new platform involves a combination of gaze and manual control. Only one hand is needed to insert and withdraw the endoscope leaving the other hand free. The other hand could be used to control instruments which currently is performed by a second person requiring the endoscopist to communicate and be reliant on assistant to perform critical tasks such as knife deployments, snare closure and coagulation grasping. When the endoscopist has the area of interest in the field of view all tip control could be done hands free with only intermittent use of the right hand to withdraw or insert the endoscope if needed during the intervention. The previous iteration was entirely hands free but introduction of the combined controlled system is demonstrated to still have a significantly improved performance and workload vs the conventional endoscope control system.

Limitations of this study include the simulated environment which does not truly represent the deformability and peristalsis seen in real endoscopy. Ethics have been obtained for a human trial which is planned next. Lower gastrointestinal endoscopy has different requirements including torque steering and therefore the outcomes cannot be applied to lower endoscopy.

## Conclusions

The remit of endoscopy continues to grow requiring precision control via the conventional despite its well documented non intuitive and poor ergonomic control system. There has been much work on modernising the control system through use of robotics. The new iteration of our gaze-control system has proved to be a more intuitive control system than conventional endoscopy and is the only system we are aware of using gaze as a control input. The tip control remains hands free but insertion is now manual. This study has shown it remains favourable as a control system to a conventional endoscope. The updated system has a significantly smaller footprint and due to its setup can be fitted to any conventional endoscope through printing different 3D gears at a low cost. This system represents a further exciting step in this novel endoscope control system with the potential to reduce mental workload associated with advanced endoscopic procedures.

## Supplementary Information

Below is the link to the electronic supplementary material.

Supplementary file1 (DOCX 16 KB)

## Data Availability

The authors confirm that the data supporting the findings of this study are available within the article and its supplementary materials. Further data available by request from the corresponding author.
