# Supplementary material for: Eye-controlled endoscopy — a benchtop trial of a novel robotic steering platform — iGAZE2
Source: J Robot Surg. 2024 Jun 25;18(1):266. doi: 10.1007/s11701-024-02022-5 (PMC11199204; doi:10.1007/s11701-024-02022-5)
Supplement: Supplementary file 1 — Supplementary file1 (DOCX 16 KB) [file 11701_2024_2022_MOESM1_ESM.docx]

**Supplementary Material**

NASA TLX scores all users

|  | Eye Gaze | | | | | Total Eye Gaze | Total Scope | Scope | | | | |
| --- | --- | --- | --- | --- | --- | --- | --- | --- | --- | --- | --- | --- |
| User | MD | PD | TD | EF | FR |  |  | MD | PD | TD | EF | FR |
| 1 | 20 | 15 | 20 | 20 | 50 | 125 | 260 | 35 | 50 | 70 | 60 | 45 |
| 2 | 40 | 15 | 25 | 55 | 55 | 190 | 260 | 20 | 60 | 70 | 65 | 45 |
| 3 | 25 | 70 | 60 | 80 | 15 | 250 | 500 | 100 | 100 | 100 | 100 | 100 |
| 4 | 55 | 10 | 40 | 40 | 50 | 195 | 240 | 25 | 85 | 40 | 45 | 45 |
| 5 | 50 | 20 | 35 | 45 | 20 | 170 | 285 | 65 | 55 | 50 | 65 | 50 |
| 6 | 15 | 15 | 15 | 30 | 15 | 90 | 300 | 60 | 20 | 65 | 65 | 90 |
| 7 | 45 | 60 | 45 | 35 | 20 | 205 | 410 | 75 | 85 | 90 | 85 | 75 |
| 8 | 50 | 10 | 25 | 20 | 5 | 110 | 420 | 100 | 95 | 70 | 100 | 55 |
| 9 | 30 | 40 | 20 | 50 | 45 | 185 | 280 | 40 | 70 | 60 | 60 | 50 |
| 10 | 25 | 5 | 10 | 5 | 5 | 50 | 255 | 70 | 20 | 65 | 70 | 30 |
| 11 | 35 | 20 | 30 | 50 | 25 | 160 | 285 | 60 | 55 | 60 | 70 | 40 |
| 12 | 45 | 15 | 35 | 50 | 50 | 195 | 340 | 70 | 65 | 65 | 75 | 65 |
